# Prevalence and Burden of Musculoskeletal Pain among Cardiac Sonographers in Eastern Province of Saudi Arabia: A Cross-Sectional Study

**DOI:** 10.3390/jcm13113184

**Published:** 2024-05-29

**Authors:** Lamia Al Saikhan, Ali M. Alshami

**Affiliations:** 1 Department of Cardiac Technology, College of Applied Medial Sciences, Imam Abdulrahman Bin Faisal University, Dammam 31441, Saudi Arabia; 2 Department of Physical Therapy, College of Applied Medial Sciences, Imam Abdulrahman Bin Faisal University, Dammam 31441, Saudi Arabia; alshami@iau.edu.sa

**Keywords:** echocardiography, ultrasound, occupational pain, musculoskeletal pain, ergonomics

## Abstract

**Background**: Cardiac sonographers are at a high risk for work-related musculoskeletal pain (WMSP), a major occupational health problem. With limited research on WMSP prevalence among this population in Saudi Arabia, this study aimed to investigate the prevalence and impact of WMSP in cardiac sonographers in the Eastern Province of Saudi Arabia compared to a control group of healthcare professionals. **Methods**: An electronic survey was administered to cardiac sonographers (study group) and other healthcare professionals (control group) exposed to different occupational hazards, including allied healthcare professionals, physicians, and nurses. Modified versions of the Nordic, QuickDASH, and QuickDASH work questionnaires were used. The χ^2^ test was performed for comparisons. **Results**: A total of 168 participants completed the survey (mean age: 31.6 ± 7.7 years). Among them, 127 (76.1%) were females, comprising 61 (36.3%) sonographers and 107 (63.7%) controls. Overall, WMSP was more common (82% versus 65%, *p* = 0.020) and severe (*p* = 0.041) in cardiac sonographers than in controls. The most affected body regions in cardiac sonographers were the shoulders (72.0% versus 29.0%), followed by the hands (56.0% versus 24.6%), compared to those of the control participants. Pain experienced by cardiac sonographers significantly interfered with social and work-related activities (*p* < 0.05 for all). A higher number of cardiac sonographers planned to change their profession than control participants (41% versus 15.2%; *p* < 0.0001) owing to pain. **Conclusions**: WMSP was more common and severe in cardiac sonographers than in control participants of other healthcare professions in the Eastern province of Saudi Arabia and interfered significantly with their social and work-related activities and future employment plans. Therefore, preventive interventional studies are required in the future.

## 1. Introduction

Work-related musculoskeletal pain (WMSP) is considered a major occupational health problem associated with the cardiac sonography profession compared to other diagnostic medical ultrasound specialties [1]. Echocardiography-related demands include maintaining awkward postures with tight imaging windows, limited movement, and constant handgrip pressure, which collectively increase the risk of WMSP [1,2]. This pain adversely affects sonographers’ quality of life, physical health, and well-being [3,4,5,6]. It is also a common cause of work restrictions, sickness, and absenteeism owing to occupational injuries, imposing additional financial implications on employers [3,4,5,6].

Past research confirms the substantial burden of WMSP on cardiac sonographers [2,7,8]. However, most studies fail to compare these findings with other professions (i.e., a comparision control group), overlook awareness needs, or neglect to address future impacts and preventative practices [2,3,9]. Existing research has gaps in comprehensively characterizing WMSP among cardiac sonographers compared to other healthcare professionals. This includes a lack of recent data on associated symptoms, working conditions, and potential risks.

In Saudi Arabia, the field of cardiac sonography has recently emerged, particulary in the Eastern Province of Saudi Arabia with the establishment of the first educational program in Saudi Arabia in 2008/2009 [10]. As the number of graduates from this program has increased in the region, concerns about WMSP have arisen. This is particularly important because the program predominantly consists of females, and research indicates a strong association between female and experiencing WMSP [10,11]. Consequently, this demand is anticipated to rise further, with the increasing burden of cardiovascular risk factors among the Saudi Arabian population [12,13,14].

The burden of WMSP among cardiac sonographers in Saudi Arabia has not been thoroughly studied, and the scanning conditions and/or techniques under which cardiac sonographers work may differ regionally [15]. To date, only one study has comprehensively investigated the prevalence, characteristics, consequences, and awareness of WMSP among cardiac sonographers in Saudi Arabia and found that WMSP is highly prevalent among cardiac sonographers [11]. Therefore, this study aimed to investigate the prevalence and impact of WMSP among cardiac sonographers in the Eastern province of Saudi Arabia.

## 2. Materials and Methods

### 2.1. Study Setting and Design

This cross-sectional study was approved by the Institutional Review Board of Imam Abdulrahman Bin Faisal University (IRB-2022-03-235; approval date: 15 June 2022). This study was conducted in accordance with the principles of the Declaration of Helsinki and followed the Strengthening the Reporting of Observational Studies in Epidemiology (STROBE). All the participants provided written informed consent before participating in the study. The data were collected from the Eastern Province of Saudi Arabia. Individuals performing cardiac sonography were included in the study (study group), regardless of whether they also performed other types of sonography. Another group of non-sonographers (i.e., other healthcare professionals) exposed to different occupational hazards was recruited as a control group. The control group consisted of healthcare professionals who were employed full time at the time of the study.

### 2.2. Survey

To ensure sample representativeness of cardiac sonographers in the Eastern province, the survey was distributed electronically via Google Forms by Saudi healthcare professional groups, with an invitation letter for participants that included details of the study. The survey was also sent to the official representatives of the Saudi Society of Cardiovascular Technologists and the Saudi Arabian Society of Echocardiography to circulate the survey among their members. The survey was further broadcast via social media platforms, including LinkedIn, Twitter, WhatsApp, and alumni network groups, with the aim of reaching more potential participants. The survey was conducted between July and October 2022. Voluntary and anonymous participation was sought to complete the survey.

The survey used in this study was based on established and validated questionnaires in the literature [7,16,17,18,19], including the modified Nordic questionnaire (designed by the Mayo Clinic Survey Center) [7], the QuickDASH questionnaire [16], and the QuickDASH Work Questionnaire [17]. The survey consisted of 45 questions divided into the following sections. Section one captured demographics (10 questions), including occupation, age, sex, nationality, education, height, weight, body mass index (BMI), exercise outside work, including accumulated duration per week, type of exercise, and handedness.

Section two captured the work assignments of cardiac sonographers (six questions), including the number and duration of assigned echocardiographic scans per day, the number and duration of breaks during the workday, and whether exam or task rotation was considered in the workplace.

Section three captured work-related activities (nine questions), including years of experience in the current position, work settings, scanning hand and position (i.e., ergonomics), number of scanning hours per day, and workload.

Section four captured the musculoskeletal pain characteristics and symptoms (20 questions) using the modified Nordic questionnaire [7]. Musculoskeletal pain was defined as the pain or discomfort experienced in the past 12 months resulting from work-related activities. Additional questions related to the WMSP (location, severity, duration, progression, and aggravating and alleviating factors) were asked if the participant reported musculoskeletal pain. Pain severity was assessed on a scale of 0–10 (0 = no pain at all, 10 = worst imaginable pain). The duration of pain symptoms was defined as follows: seldom (1–7 days), sometimes (8–30 days), frequently (more than 30 days but not every day), and always (every day). Aggravating and alleviating factors were assessed by asking whether cardiac sonographers regularly alternated scanning hands, regularly relaxed their handgrip for a few seconds while scanning, regularly engaged in stretching between patients, tended to perform high-pressure handgrip while scanning, and/or tended to incline toward the right side while scanning. Furthermore, the impact of WMSP was assessed as follows: (1) seeking medical evaluation, being diagnosed with a disease related to scanning, or receiving medical treatment including surgical treatment; (2) interruption of work because of musculoskeletal pain, including missing workdays, changing work-related responsibilities, and planning to change profession; and (3) pain associated with interference in performing daily, recreational, and work-related activities. The QuickDASH and QuickDASH-work questionnaires were sent to participants to assess their physical function and symptoms of the upper limb and to evaluate their ability to perform work activities, respectively. The former uses 11 items, and the latter uses four items, each of which is scored from 1 to 5. A total score of 100 was calculated, where 100 indicated highest degree of disability [16,17].

The first version of the tool was evaluated for content clarity and ambiguity by conducting a pilot test among 10 cardiac sonographers and healthcare professionals before being formally distributed. Amendments were made based on the feedback and comments received to further improve the design of the electronic survey. No major problems were identified, and hence, the final version was approved.

### 2.3. Statistical Analysis

Continuous variables are expressed as mean ± SD or median (interquartile range) if skewed. Categorical variables are expressed as counts (percentages). The following comparisons were considered: (1) cardiac sonographers versus control participants (overall sample) and (2) cardiac sonographers with pain versus control participants with pain. Continuous variables were compared using a two-sample *t*-test with unequal variance, if necessary (Welch–Satterthwaite *t*-test), or the Wilcoxon rank-sum test, as appropriate. Categorical variables were compared using χ^2^ test or Fisher’s exact test as appropriate. Statistical significance was defined as a two-tailed *p*-value < 0.05. All statistical analyses were performed using STATA version 15.1 (StataCorp, LLC, Houston, TX, USA).

The minimum required sample size was determined to be at least five times the number of the questionnaire items provided that the sample size is ≥100 participants [20]. Thus, 100 participants were required to achieve a statistical power of 80%, based on the questionnaire items of section four. Considering a dropout rate of 20%, 120 were at least required to be recruited to complete the survey.

## 3. Results

### 3.1. Participants’ Characteristics

A total of 169 participants completed the survey; however, one participant was excluded from the analysis as they did not agree to participate in the study. Of the 168 participants, 61 (36.3%) sonographers and 107 (63.7%) controls were included in the analysis. The mean age of the total sample was 31.6 ± 7.7 years, 127 (76.1%) were women, and 129 (77.3%) were Saudi citizens. Among the sonographers, only 58 (95%) had performed cardiac sonography. Among the control participants, 37% were allied healthcare professionals, 21% physicians, 28% nurses, and 14% other healthcare professionals.

Table 1 summarizes the characteristics of the two groups. Compared to the control participants, cardiac sonographers were younger and had lower BMI values. There were more Saudis and females among the cardiac sonographers than the control participants. Most cardiac sonographers were bachelor’s degree holders, worked between 8 and 9 h per day, and had ≤5 years of experience. The control participants were more likely to have more lunch breaks, exam/task rotation, and additional responsibilities. Headache was the most common medical condition in both groups, with no evidence of a difference between them (cardiac sonographers: 37.7%; control participants: 43.9%). All other variables were similar between the two groups.

### 3.2. Work Assignments of Cardiac Sonographers

Among the cardiac sonographers, 63.4% were assigned between 5–7 and 7–9 scans per day, 91.6% spent between ≤30 min and 30–45 min per scan, 65% were seated in a chair beside the patient bed, and 43.3% devoted 4–6 h to scanning per day. In addition, 70% of the cardiac sonographers had no breaks between booked scans, 71.2% had bedside scans generally equally distributed, 51.7% performed studies in conjunction with fellows/students, only 43.3% considered using technologies such as 3D echo to reduce scanning time, 26.7 had overnight call responsibilities, and 40.7% worked on weekends (Table 2).

### 3.3. Characteristics of Musculoskeletal Pain

**Figure 1:**
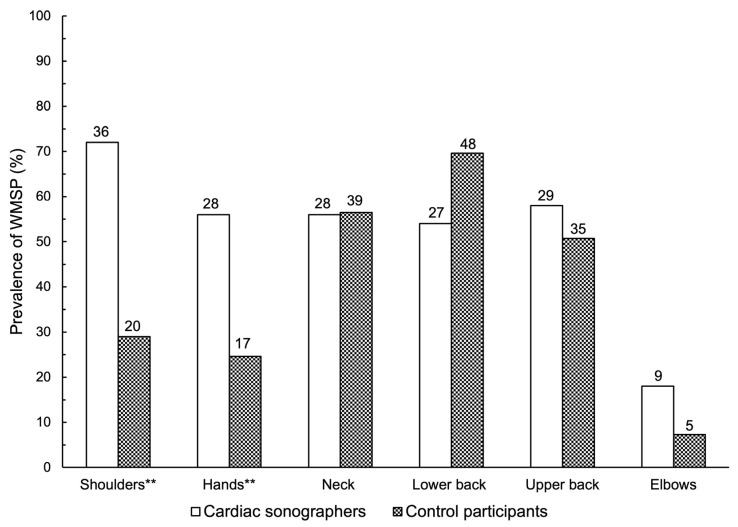
Prevalence of work-related musculoskeletal pain (WMSP) by body regions in the cardiac sonographers (*n* = 50) compared to the control participants (*n* = 69). ** *p*-value < 0.0001. Data represent percentages (%). Numbers represent frequencies.

**Figure 2:**
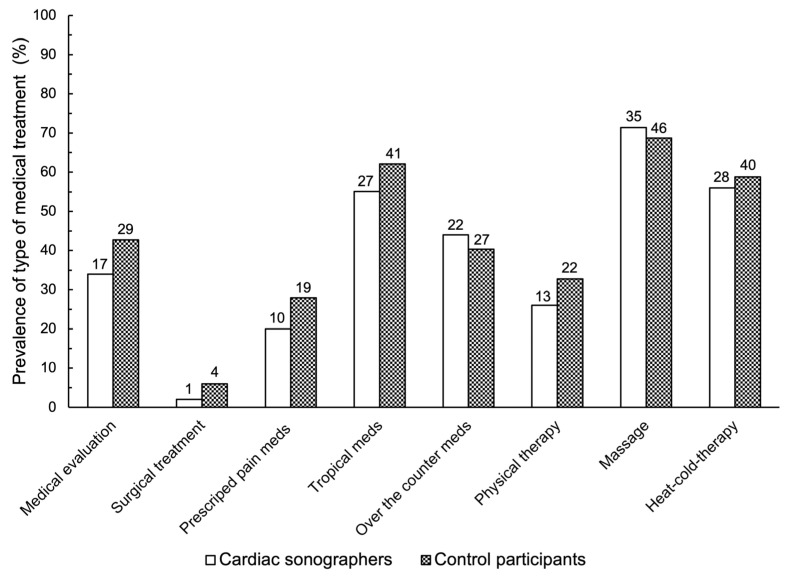
Prevalence of type of medical evaluation/treatment in the cardiac sonographers (*n* = 50) compared to the control participants (*n* = 68). *p*-value ≥ 0.292. Data represent percentages (%). Numbers represent frequencies.

Overall, pain was more prevalent in cardiac sonographers (82%) than in controls (65%) (*p* = 0.020). Interestingly, pain among cardiac sonographers was more prevalent in the shoulders (72.0% versus 29.0%), followed by the hands (56.0% versus 24.6%), than in the control participants (Figure 1). Pain was more severe in cardiac sonographers (5.8 ± 1.7/10) than in controls (5.1 ± 1.8/10) (*p* = 0.041). However, this difference did not reach the minimal clinically important difference (MCID) of 1 point [22]. Both groups were similar in terms of pain duration and progression. Both groups were similar in terms of seeking medical evaluation or receiving medical treatment (Figure 2).

Among 49 cardiac sonographers with pain, we observed that a high proportion of them tended to incline toward the right side while scanning (69.4%) and perform a high-pressure handgrip while scanning (69.4%) followed by a relaxed hand grip for a few seconds while scanning regularly (59.2%), whereas a lower proportion of cardiac sonographers with pain tended to stretch regularly between patients (exams/scans) (38.8%) and alternate scanning hands regularly (6.1%).

Table 3 summarizes the characteristics of cardiac sonographers and control participants with WMSP. Compared to control participants with WMSP, cardiac sonographers were younger and had lower BMI values. Specific medical conditions, such as neck/back arthritis, carpal tunnel syndrome, herniated disk, and spinal stenosis, were less common in both groups. Most cardiac sonographers with pain were bachelor’s degree holders, worked between 8 and 9 h per day, and had ≤5 years of experience. There were more Saudis and females among cardiac sonographers with pain than among the control participants with pain. Control participants with pain had more lunch breaks and additional tasks such as research, education, and administration. Both groups were similar in terms of frequency, type of exercise, and workplace settings.

### 3.4. Impact of Musculoskeletal Pain

Table 4 summarizes the results of the impact of WMSP among cardiac sonographers compared with control participants. Cardiac sonographers’ pain significantly interfered with their social activities and interrupted their work-related activities (*p* < 0.05 for all). A higher number of cardiac sonographers (41%) compared to control participants (15.2%) planned to change their profession owing to pain (*p* ≤ 0.0001). Cardiac sonographers had higher QuickDASH and QuickDASH Work scores than those of control participants. However, this difference was not statistically significant.

## 4. Discussion

This is the first study to comprehensively investigate the prevalence and impact of WMSP in the Eastern Province of Saudi Arabia. The findings revealed that WMSP was more prevalent among cardiac sonographers (82%) than among control participants (65.1%) from other healthcare professions. Pain was more severe in cardiac sonographers (5.8/10) than in controls (5.1/10), although this difference was less than the MCID [22]. The shoulders and hands were the most painful body regions in cardiac sonographers compared to control participants. This pain interrupted social and work-related activities and adversely affected future employment plans.

Our findings agree with those of previous domestic and global studies that demonstrated a high prevalence of WMSP among cardiac sonographers [2,7,8,11]. This high percentage of WMSP necessitates a holistic approach despite advanced developments in the design of ergonomic workstations with sophisticated equipment and ultrasound systems. The pain locations revealed by cardiac sonographers in our study were consistent with those reported in our previous national study and in the literature [2,8,9,11]. Female sex is also a risk factor for WRMSP [23]. Indeed, we found that cardiac sonographers were mostly female, which is of particular concern because cardiac sonographers in Saudi Arabia are predominantly female [10,11]. In our previous study, we found that cardiac sonographers experiencing pain tended to perform high-pressure handgrips while scanning, inclined toward the right side, worked on weekends, and were exposed to a higher workload than those without pain [11]. Overall, the data obtained in this study agree with these findings.

In this study, we found that pain experienced by cardiac sonographers significantly interfered with their social and work-related activities, as well as their future employment plans. These findings agree with those of Barros-Gomes et al. [7] and our previous study [11], which reported adverse effects on well-being, sleep patterns, work responsibilities, and future employment plans. Taken together, these data highlight the need for the development of a culture of safety in any echocardiography department, which can be regarded as one of the most impactful approaches for reducing injuries in any process [1,5,24].

Our study’s questionnaire holds promise for various clinical applications in managing WMSP among cardiac sonographers. For example, the questionnaire can serve as a quick and efficient screening tool to identify cardiac sonographers at risk for developing WMSP. Early detection allows for prompt intervention and management of symptoms. Moreover, the questionnaire can aid in assessing the severity of WMSP symptoms experienced by cardiac sonographers. This information can guide personalized treatment plans and monitor treatment progress. In addition, by analysing questionnaire responses, healthcare professionals might be able to identify workplace factors or ergonomic characteristics that contribute to WMSP risk in cardiac sonographers. This knowledge can inform preventive strategies in the clinical setting. To enhance the generalizability and clinical utility of our questionnaire, we recommend its validation by conducting studies with a broader participant pool that includes a larger number of cardiac sonographers from various regions and demographics. This broader representation would ensure the instrument is applicable to a wider range of cardiac sonographers in clinical practice. Furthermore, the questionnaire can be a valuable tool for evaluating the effectiveness of interventions aimed at reducing WMSP in cardiac sonographers. Future studies could explore how the questionnaire can be used to assess changes in symptom severity and WMSP prevalence.

This study had certain limitations. Owing to the cross-sectional nature of this study, a potential cause–effect relationship between pain and its associated factors cannot be determined with certainty. Nevertheless, this study aimed to provide baseline information on the characteristics and impact of WMSP among cardiac sonographers compared with those of other healthcare professionals. In addition, the number of cardiac sonographers was small compared to that of the control participants, which could have resulted in limited power in some of the subgroup analyses. We cannot exclude the possibility that employees could not accurately recall their previous experiences, causing potential over- or under-estimation, although our findings were consistent with the most current literature.

## 5. Conclusions

The prevalence and severity of WMSP were higher in cardiac sonographers than in control participants from other healthcare professionals in the Eastern province of Saudi Arabia. Pain was most commonly experienced in the shoulders and hands. This pain in cardiac sonographers has significantly interfered with their social activities, interrupted their work-related activities, and adversely affected their future employment plans. Our findings confirm the need to design preventive ergonomic strategies and/or interventions to prevent WMSP and/or minimize employee disability and career-ending injuries among cardiac sonographers.

## Figures and Tables

**Table 1:** Characteristics of participants in the study groups.

Variable	Cardiac Sonographers (*n* = 61)	Control Participants (*n* = 107)	*p-*Value
Age (year)	28.0 ± 5.4	33.6 ± 8.1	<0.0001
Sex, Female	56 (93.3)	71 (66.4)	<0.0001
Nationality, Saudi	59 (96.7)	70 (66.0)	<0.0001
Body mass index (kg/m^2^)	23.4 ± 4.0	25.1 ± 4.4	0.014
Education			0.001
Diploma	2 (3.3)	14 (13.1)	
Bachelor	56 (91.8)	64 (59.8)	
Masters	1 (1.6)	10 (9.3)	
PhD	1 (1.6)	13 (12.2)	
Others	1 (1.6)	6 (5.6)	
Regular exercise *			0.544
No/seldom	30 (49.2)	43 (40.6)	
Once per week	13 (21.3)	28 (26.4)	
At least 3 times per week	18 (29.5)	35 (33.0)	
Type of exercise			
Weightlifting	11 (18.0)	22 (20.6)	0.692
Stretching/Pilates	11 (18.0)	14 (13.1)	0.386
Aerobic	21 (34.4)	51 (47.7)	0.095
Yoga	8 (13.1)	8 (7.5)	0.231
Others	4 (6.6)	17 (15.9)	0.079
Medical conditions			
Carpal tunnel syndrome	4 (6.6)	8 (7.7)	0.786
Neck/back arthritis	3 (4.9)	5 (4.7)	0.943
Herniated disk	2 (3.3)	5 (4.7)	0.664
Spinal stenosis	2 (3.3)	4 (3.7)	0.877
Headaches	23 (37.7)	47 (43.9)	0.432
Handedness			0.468
Right	56 (91.8)	102 (96.2)	
Left	4 (6.6)	3 (2.8)	
Ambidextrous	1 (1.6)	1 (0.9)	
Work setting			0.071
Public hospital	33 (54.1)	62 (58.5)	
Private hospital	25 (41.0)	31 (29.3)	
Private outpatient clinic	3 (4.9)	4 (3.8)	
Others	0 (0)	9 (8.5)	
Years in current position			<0.0001
<1	14 (23.0)	16 (15.0)	
1–5	35 (57.4)	32 (29.9)	
>5–10	7 (11.5)	27 (25.2)	
>10–15	2 (3.3)	19 (17.8)	
>15	3 (4.9)	13 (12.2)	
Working hours per day			0.002
7	3 (4.9)	13 (12.2)	
8	31 (50.8)	51 (46.7)	
9	25 (41.0)	23 (21.5)	
10	2 (3.3)	20 (18.7)	
Lunch break	38 (63.3)	86 (80.4)	0.016
Lunch break duration			0.272
≤30 min	24 (49.0)	35 (37.2)	
30–45 min	8 (16.3)	24 (25.5)	
45–60 min	5 (10.2)	13 (13.8)	
60 min	12 (24.5)	18 (19.2)	
≥60 min	0 (0)	4 (4.3)	
Task/exam rotation	35 (57.4)	76 (72.4)	0.048
Additional education, research, or administrative responsibilities	20 (32.8)	60 (55.1)	0.004

Data are expressed as count (%), except for age and body mass index (Mean ± SD). * Regular exercise was defined as planned physical activity performed consistently to improve or maintain physical fitness and overall health [21].

**Table 2:** Work assignment of cardiac sonographers.

Assigned Scans Per Day, (*n* = 60)	*n* (%)
≤5	15 (25)
5–7	19 (31.7)
7–9	19 (31.7)
≥10	7 (11.7)
Average time per scan, (*n* = 60)	
≤30 min	26 (43.3)
30–45 min	29 (48.3)
45–60 min	5 (8.3)
≥60 min	0 (0)
Analysis performed during scanning time (*n* = 59)	48 (81.4)
Primarily scanning hand (*n* = 60)	
Right	57 (95)
Left	3 (5.0)
Ambidextrous	0 (0)
Scanning position (*n* = 60)	
Sitting in a chair next to the patient bed	39 (65)
Sitting on a bed	0 (0)
Standing	3 (5)
Alternating sitting and standing	18 (30)
Scanning hours per day (*n* = 60)	
≤4	16 (26.7)
4–6	26 (43.3)
6–8	17 (28.3)
≥8	1 (1.7)
Breaks between booked scans (*n* = 59)	18 (30.5)
Overnight call (*n* = 60)	16 (26.7)
Work weekends (*n* = 59)	24 (40.7)
Bedside scans generally equally distributed (*n* = 59)	42 (71.2)
Often perform studies in conjunction with fellows or student (*n* = 60)	31 (51.7)
Considers using technologies such as 3D echo to reduce scanning time (*n* = 60)	26 (43.3)

Data are expressed as count (%).

**Table 3:** Characteristics of cardiac sonographers and control participants with musculoskeletal pain.

Variable	Cardiac Sonographers (*n* = 50)	Control Participants (*n* = 69)	*p-*Value
Age (year)	27.7 ± 4.1	33.7 ± 8.1	<0.0001
Sex, Female	49 (98)	48 (67.6)	<0.0001
Nationality, Saudi	48 (96)	42 (60.9)	<0.0001
Body mass index (kg/m^2^)	23.3 ± 4.1	25.3 ± 4.5	0.015
Education			0.004
Diploma	1 (2)	7 (10.1)	
Bachelor	48 (96)	46 (66.7)	
Masters	1 (2)	6 (8.7)	
PhD	0 (0)	7 (10.1)	
Other	0 (0)	3 (4.4)	
Handedness			0.503
Right	46 (92)	64 (94.1)	
Left	4 (8)	3 (4.4)	
Ambidextrous	0 (0)	1 (1.5)	
Regular exercise			0.772
No/seldom	25 (50)	30 (43.5)	
Once per week	12 (24)	18 (26.1)	
At least 3 times per week	13 (26)	21 (30.4)	
Work setting			0.038
Public hospital	26 (52)	38 (55.1)	
Private hospital	23 (46)	20 (29)	
Private outpatient clinic	1 (2)	4 (5.8)	
Other	0 (0)	7 (10.1)	
Years in current profession			0.002
<1 year	10 (20)	12 (17.4)	
1–5 years	31 (62)	20 (29)	
>5–10 years	5 (10)	18 (26.1)	
>10–15 years	2 (4)	11 (15.9)	
>15 years	2 (4)	8 (11.6)	
Total number of working hours/day			<0.0001
7	1 (2)	10 (14.5)	
8	26 (52)	30 (43.5)	
9	22 (44)	14 (20.3)	
10	1 (2)	15 (21.7)	
Lunch break	28 (57.1)	56 (81.2)	0.005
Lunch break duration			0.726
≤30 min	19 (50)	25 (41)	
30–45 min	6 (15.8)	12 (19.7)	
45–60 min	5 (13.2)	10 (16.4)	
60 min	8 (21.1)	12 (19.7)	
≥60 min	0 (0)	2 (3.3)	
Exam/task rotation is possible	32 (64)	53 (76.8)	0.126
Have additional research, education, or administrative responsibilities	14 (28)	38 (55.1)	0.003
Carpal tunnel syndrome	2 (4)	5 (7.6)	0.423
Neck/back arthritis, spinal stenosis, or herniated disk	6 (12)	7 (10.3)	0.770
Headaches	23 (46)	33 (47.8)	0.844

Data are expressed as count (%), except for age and body mass index (Mean ± SD).

**Table 4:** Impact of musculoskeletal pain in the study groups.

Variable	Cardiac Sonographers (*n* = 61)	Control Participants (*n* = 107)	*p-*Value
Due to the pain, I			
Missed work (stay at home)	17 (27.9)	26 (24.5)	0.635
Had changes in my work-related responsibilities	7 (11.7)	11 (10.3)	0.782
Was transferred to another work area (research, admin, etc.)	5 (8.2)	6 (6.0)	0.592
Had work restrictions	7 (11.5)	7 (6.9)	0.309
Had plans to change professions	25 (41.0)	16 (15.2)	<0.0001
Pain during household chores (e.g., wash walls, wash floors)			0.636
Unable	1 (1.7)	2 (2.1)	
Severe difficulty	0 (0.0)	3 (3.2)	
Moderate difficulty	8 (13.3)	9 (9.5)	
Mild difficulty	18 (30.0)	31 (32.6)	
No difficulty	33 (55.0)	50 (52.6)	
Pain during recreational activities			0.465
Unable	0 (0)	2 (2.2)	
Severe difficulty	2 (3.4)	1 (1.1)	
Moderate difficulty	5 (8.5)	5 (5.4)	
Mild difficulty	14 (23.7)	29 (31.2)	
No difficulty	38 (64.4)	56 (60.2)	
Pain interfered with social activities with family/friends/neighbors/groups			0.035
Extremely	1 (1.7)	1 (1.1)	
Quite a bit	0 (0.0)	4 (4.2)	
Moderately	2 (3.3)	12 (12.6)	
Slightly	24 (40.0)	21 (22.1)	
Not at all	33 (55.0)	57 (60.0)	
Limited in work or other regular daily activities due to the pain			0.956
Unable	0 (0.0)	0 (0)	
Very limited	1 (1.7)	1 (1.1)	
Moderately limited	4 (6.8)	8 (8.4)	
Slightly limited	13 (22.0)	19 (20.0)	
Not limited at all	41 (69.5)	67 (70.5)	
Difficulty sleeping due to the pain			0.529
Unable	0 (0)	1 (1.0)	
Severe difficulty	1 (1.7)	1 (1.0)	
Moderate difficulty	3 (5.0)	9 (9.3)	
Mild difficulty	21 (35.0)	24 (24.7)	
No difficulty	35 (58.3)	62 (63.9)	
Difficulty using usual technique for work due to the pain			0.115
Unable	0 (0.0)	0 (0)	
Severe difficulty	1 (1.7)	1 (1.1)	
Moderate difficulty	0 (0.0)	4 (4.3)	
Mild difficulty	19 (31.7)	17 (18.3)	
No difficulty	40 (66.7)	71 (76.3)	
Spending your usual amount of time doing work			0.039
Unable	0 (0)	0 (0)	
Severe difficulty	1 (1.7)	1 (1.1)	
Moderate difficulty	0 (0)	8 (8.4)	
Mild difficulty	22 (37.3)	21 (22.1)	
No difficulty	36 (61.0)	65 (68.4)	
Doing your work as you would like			0.009
Unable	0 (0)	0 (0)	
Severe difficulty	0 (0)	1 (1.1)	
Moderate difficulty	7 (11.7)	6 (6.4)	
Mild difficulty	21 (35.0)	14 (14.9)	
No difficulty	32 (53.3)	73 (77.7)	
Difficulty doing usual work due to the pain			0.505
Unable	0 (0)	0 (0)	
Severe difficulty	1 (1.7)	1 (1.1)	
Moderate difficulty	2 (3.4)	4 (4.3)	
Mild difficulty	23 (39.0)	26 (27.7)	
No difficulty	33 (55.9)	63 (67.0)	
QuickDASH score	11.4 (4.5–18.2) (*n* = 56)	6.8 (2.3–15.9) (*n* = 88)	0.095
QuickDASH Work score	12.5 (0–18.75) (*n* = 58)	0 (0–12.5) (*n* = 93)	0.055

Data are expressed as count (%), except for QuickDASH scores (Median (interquartile range).

## Data Availability

The original contributions presented in the study are included in the article/supplementary material, further inquiries can be directed to the corresponding author/s.
